# Integrating environmental sustainability into food-based dietary guidelines in the Nordic countries

**DOI:** 10.29219/fnr.v68.10792

**Published:** 2024-10-25

**Authors:** Ellen Trolle, Jelena Meinilä, Hanna Eneroth, Helle Margrete Meltzer, Inga Þórsdóttir, Thorhallur Halldorsson, Maijaliisa Erkkola

**Affiliations:** 1 National Food Institute, Technical University of Denmark, Kgs. Lyngby, Denmark; 2 Department of Food and Nutrition, University of Helsinki, Helsinki, Finland; 3 Department of Risk Benefit Assessment, Swedish Food Agency, Uppsala, Sweden and Department of Energy and Technology, Swedish University of Agricultural Sciences, Uppsala, Sweden; 4 Norwegian Institute of Public Health, Oslo, Norway; 5 Faculty of Food Science and Nutrition and Unit for Nutrition Research at the Health Science Institute, School of Health Sciences, University of Iceland

**Keywords:** diet, plant-based, animal-based, environmental impact, dietary pattern recommendation, nutrition, scoping review, scenario

## Abstract

The overall aim of this paper was to provide background knowledge to the Nordic Nutrition Recommendations 2023 Committee for integrating environmental sustainability in a framework for national Food-Based Dietary Guidelines (FBDG) within the Nordics and Baltics. Additionally, this paper aims to give an overview of recent Nordic scientific literature on environmental impact of foods and dietary patterns and of the FBDG of the Nordics. Finally, we suggest methods for developing national sustainable FBDG. Nordic and Baltic studies on sustainability of diets were searched in August 2022 and complemented with additional relevant literature. The studies show that current diets are far from environmentally sustainable, exceeding planetary boundaries for most impact categories; meat and dairy products being the largest contributors to dietary greenhouse gas emissions (GHGEs) and land use. Scenario, modelling, optimisation and intervention studies confirm the potential of shifting towards more plant-based diets to improve overall diet quality in terms of both health and environmental sustainability. Such diets comprised of vegetables, fruits, legumes, potatoes, whole grain and refined cereal products, nuts, seeds and vegetable oils, with animal foods in moderate or limited amounts. The FBDG in the Nordics promotes more plant-based diets than the current average diet but could improve from further integration of environmental sustainability. To form basis for sustainable FBDG dietary modelling at the national level, prioritising health outcomes and nutritional adequacy is essential. Second, integrating environmental sustainability involves estimating the impact of food choices and amounts on GHGE, land and water use, eutrophication and biodiversity loss. Exploring positive and negative implications of fortified foods and supplementation in relation to nutrient intake, health and environmental sustainability may be needed. Implementing dietary transition requires solutions beyond FBDG to ensure affordability, acceptability and ease of adaption.

## Popular scientific summary

- Nordic studies indicate that current diets are environmentally unsustainable, with meat and dairy products being the primary contributors to dietary greenhouse gas emissions (GHGEs) and land use.
- A shift towards more plant-based diets, with animal foods consumed in moderate or limited amounts, improves overall diet quality in terms of both health and environmental sustainability.
- Dietary modelling at national level should form basis for determining amounts of specific foods and food groups in future sustainable FBDG.

## Concepts

Blue and green water: ‘Blue water’ is the water in rivers, lakes and ground water. ‘Green water’ is the water that feeds the system as rain and forms soil moisture that is absorbed by plants (and then exhaled as vapour flow).

Discretionary foods: Discretionary foods are foods that are not essential for our health. They are characterised by being high in saturated fat, added sugars, salt and/or alcohol.

Extinction rate: A measure for biodiversity loss E/MSY = extinctions per million species–years, extinction being the dying out of a species, see more details in Moberg et al. (2020). PDF (Potential Disappeared Fraction) is another measure for biodiversity loss, see Kyttä et al. (2023).

Plant-based diet: An umbrella term, here defined as dietary patterns with a focus on foods primarily derived from plants. These foods include a high variety of vegetables, fruit and berries, cereal products, vegetable oils, legumes (pulses), and nuts and seeds. The diets may contain moderate to low amounts of animal-sourced food such as fish, dairy, eggs and meat.

The umbrella term encompasses plant-rich diets and vegetarian diets and vegan diets, defined as follows:

Plant-rich diets (may also be referred to as flexitarian diets): They are high in a variety of vegetables, fruit and berries, cereal products, vegetable oils and fats, legumes, and nuts and seeds. The diets contain low or moderate amounts of animal-sourced foods such as fish, dairy, eggs and meat.

Vegetarian diet (sometimes referred to as lacto-ovo vegetarian): Includes eggs and dairy foods, but no meat, poultry, fish or seafood.

Vegan diet: Includes no animal-sourced foods.

The Baltics: The three Baltic countries = Estonia, Latvia and Lithuania

The Nordics: The five Nordic countries = Denmark, Finland, Iceland, Norway and Sweden.

## Introduction

Climate change is amongst the most serious environmental crises, alongside biodiversity loss and degradation of ecosystems, partly resulting from deforestation and eutrophication. Food production and consumption are major drivers of these impacts (1, 2).

Nutritional adequacy and health aspects have been the main objectives of the development of national Food-Based Dietary Guidelines (FBDG) since the first guidelines on the food composition of the diet were introduced in the Nordic countries in the beginning of the 1900s, for example, in Denmark during the first world war (3). FBDG in the Nordic countries further developed as the nutrition science evolved to recognise the role and function of micronutrients and the evidence for the relationship between food intake and disease risk. Providing complementary advice on environmental aspects to health-based FBDG was first introduced in Sweden in 2008 (4). In 2010, sustainable diets were defined by The Food and Agriculture Organization (FAO) as diets ‘with low environmental impacts which contribute to food and nutrition security and to healthy life for present and future generations. Sustainable diets are protective and respectful of biodiversity and ecosystems, culturally acceptable, accessible, economically fair and affordable; nutritionally adequate, safe and healthy, whilst optimising natural and human resources’ (5). Since then, work in this field has developed both in the Nordic countries and worldwide. The Nordic Nutrition Recommendations 2012 (6) included a chapter about sustainable food consumption and environmental issues. FAO and the World Health Organization (WHO) have developed Guiding Principles for Sustainable Healthy Diets (7). They are to be used by governments and other actors in policymaking and communications and to be further translated into clear, non-technical information and messaging such as official national FBDG. The Guiding Principles for Sustainable Healthy Diets are food-based and take nutrient recommendations into account, whilst considering environmental, social/cultural and economic sustainability. One of the guiding principles (#14) advises taking current food consumption patterns into account. Calculating the environmental impact, nutritional adequacy and health impact of the whole diet – in different population groups, with different dietary specifications, such as vegetarian or flexitarian diets – is important in providing knowledge about future demands on food supply and food systems.

The overall aim of this paper was to provide background knowledge to be used by the NNR2023 Committee when integrating environmental sustainability in a framework for FBDG within the Nordics and Baltics. The specific objectives were to provide an overview of the most recent work in the Nordics on the environmental impact of foods and dietary patterns and on the development of FBDG from the viewpoint of health and environmental sustainability and, finally, to suggest methods for the development of national sustainable FBDG in the Nordic and Baltic countries.

Together with four other papers (8–11), this paper was written in preparation for integration of sustainability into the NNR2023 report (12) (Box 1).

## Method

This paper is based on scientific literature regarding Nordic and Baltic studies on the sustainability of diets retrieved from a literature search (see Appendix A), complemented with snowball searches (literature, white papers, international and national reports). Furthermore, the current Nordic national FBDG and their scientific background are presented. Additional searches for Nordic data on sustainability of foods and methods used in developing sustainable FBDG were performed. The studies on environmental impacts of dietary patterns in Nordic countries cited in the paper are summarised in Table A1, Appendix A. This paper was in public consultation via the NNR2023 website and was subsequently revised before being peer reviewed.

All estimates of country-specific environmental impacts of dietary patterns depend on the specific data sources and how the system boundaries are handled (see Appendix B).

Box 1. *Background papers for Nordic Nutrition Recommendations 2023*

- This paper is one of many scoping reviews commissioned as part of the Nordic Nutrition Recommendations 2023 (NNR2023) project (12).
- The papers are included in the extended NNR2023 report, but, for transparency, these scoping reviews are also published in Food & Nutrition Research.
- The scoping reviews have been peer reviewed by independent experts in the research field according to the standard procedures of the journal.
- The scoping reviews have also been subjected to public consultations (see report to be published by the NNR2023 project).
- The NNR2023 committee has served as the editorial board.
- Whilst these papers are a main fundament, the NNR2023 committee has the sole responsibility for setting the final dietary reference values in the NNR2023 project.

## Environmental sustainability of dietary patterns in the Nordics

### Current diets in the Nordics

In the Nordics, the latest national dietary surveys show that the target amounts in current FBDG for meat, vegetable and fruit consumption are still far from being reached, especially by men (13). The mean daily intake of meat and meat products varies roughly between 100 and 172 g/day (cooked weight), with higher reported intakes in men than in women (some of the differences may result from variation in dietary surveying, food grouping and calculation procedures in each country). In contrast, the consumption of some plant-based protein sources, such as legumes and nuts, is low, grains/flour being the most important plant protein provider. Country-specific features include high pork consumption in Denmark, high lamb and fish consumption in Iceland, high milk consumption in Finland and Sweden and relatively high fish consumption in Norway (9, 10, 13). All countries have an excessive consumption of discretionary foods rich in sugar, saturated fat and sodium (13). The main micronutrients low in the diets of the Nordic populations are vitamin D and folate, and for some countries also selenium and potassium. In women, also the average intake of iron as well as iodine is commonly low (13). Food consumption varies according to sociodemographic groups (14–23). These include income level, education level, gender, family structure and place of residence.

### Environmental footprint of the current dietary patterns in the Nordic countries

Numerous studies have focused on the greenhouse gas emissions (GHGEs), also called climate impact or carbon footprint, to assess the environmental sustainability impact of food consumption. This is also evident in the Nordic studies that are currently available. In recent Nordic studies, the GHGE of average diets, estimated from nationally representative samples, is typically in the range of 1,500–2,200 kg CO_2_-eq. (carbon dioxide equivalents)/person/year. For example, 2,000 kg CO_2_-eq./person (56–95 years old)/year in Sweden was estimated by Hallström et al. (24), 2,200 kg CO_2_-eq./person/year in Finland by Saarinen et al. (25) and 2,040 kg CO_2_-eq./person/year in Iceland (26). A study from Norway (27) found an impact of the diet based on the Norwegian national dietary survey ‘Norkost 3’ and 2,465 kg CO_2_-eq/person/year, which was referred to as somewhat higher than an earlier estimate based on household food consumption data. In two Danish studies, GHGE of the diet was adjusted to a certain energy intake per day. Trolle et al. (28) reported 4.4 kg CO_2_-eq./10 MJ (2,390 kcal) for the average Danish diet in the national dietary survey 2001–2013, whilst Bruno et al. (29) reported 4.4 kg CO_2_-eq./2,000 kcal (8.36 MJ) based on food-balance sheets of Danish food consumption. Environmental impact estimates may differ not only due to varying food consumption patterns between the different Nordic countries and from different years and population groups, but also due to differing methodologies employed, for example, in the method of retrieving food consumption data, choice of environmental footprint databases and system boundaries of the data (9), see also Appendix B.

Two recent studies by Moberg et al. and Hallström et al. (30, 31) assessed GHGE as well as cropland use, nitrogen application, phosphorus application, consumptive water use and extinction rate of Swedish food consumption in relation to the global environmental boundaries set by the EAT-Lancet Commission (32). Based on different food consumption data (food supply data for the Swedish average diet and self-reported food intake from cohorts of age 56–95 years), they found that the food consumption exceeded all but one planetary boundary (freshwater use), exceeding the boundaries for GHGE, cropland use and application of nitrogen and phosphorus by two- to more than fourfold. The biodiversity boundary was exceeded by about fourfold (31) or sixfold (30). The global boundaries were sometimes found to be too coarse to capture local impacts, such as the impact of eutrophication (30). As the average magnitude of climate impact of food consumption in the Nordic countries seems to be similar, food consumption in all Nordic countries most likely exceeds at least the planetary boundary of climate impact as stated in a global comparison by Springmann et al. (33).

#### Impact from food groups in the Nordic diets

Amongst the foods in the Nordic diets, meat and dairy have the largest contribution to the GHGE and several other environmental impacts (25, 30). Moberg et al. showed that for GHGE as well as for cropland use, nitrogen application, phosphorus application, consumptive water use and extinction rate of Swedish food consumption, meat contributed 25–45% of the environmental impacts (30). Similar results were shown by Mogensen et al. investigating GHGE using self-selected dietary patterns based on the Danish national dietary survey in 2005–2008 (34). Relative to the three data-driven patterns (Principal Component Analysis based) of ‘green’, ‘traditional’ and ‘fast food’ pattern, with a beef content 20–28 g/10 MJ (cooked weight), a ‘high-beef’ pattern identified in a researcher-driven analysis (50 g/10 MJ, cooked weight) had 12–19% higher GHGE and required 10–20% more land area (34). Also, in a Finnish study by Meinilä et al., assessing self-selected food purchase patterns (derived by factor-analysis) and related carbon footprints, the plant-based purchase pattern had the lowest GHGE amongst the studied purchase patterns (35). For those who strongly adhered to the plant-based pattern (top 10% of the plant-based pattern score), the GHGE of the food purchases was 2,400 kg CO_2_-eq./person/year, which was 13% lower than the average GHGE (2,750 kg CO_2_-eq./person/year) and 27% lower than those in the highest 10% of the animal-based pattern score (35).

Vieux et al. studied the composition of self-selected diets that are nutritionally adequate and have low environmental impact in five European countries including Finland and Sweden (36). They found that the carbon footprint of the cluster with a good compromise between nutritional quality and dietary GHGE, ‘the more sustainable diet’, was approximately 3.8 kg CO_2_-eq./person/day (1,390 kg CO_2_-eq./person/year) (36). It was 15% lower than the average of observed diets and 45% lower than the cluster with the highest dietary GHGE. The mean age in this cluster was 46 years, the majority (62%) were women and a total of 25% of the Finnish participants and 20% of the Swedish participants were in this cluster. The GHGE of the cluster with the lowest GHGE was 21% lower than the average of observed diets and had the lowest nutritional quality. ‘The more sustainable diet’ was high in plant-based foods: approximately 700 g/day of plant foods (vegetables and fruits, legumes, nuts and seeds, starchy foods and grains), 100 g/day of meat, fish or eggs, 250 g/day of dairy, 250 g/day of composite dishes and 220 g/day of beverages and miscellaneous foods (36)

In the study of Mertens et al., the contribution of animal-sourced foods to total energy intake in an average Danish diet was 29%, but their contribution to GHGE was 64% (37). In comparison, the contribution of plant-sourced foods (legumes, grains, fruits and vegetables) to total energy intake was 60%, and their contribution to GHGE was 19% (37). In a Swedish study (31), the share of animal-based foods of the total GHGE of the diet was 71% (for both men and women) and of plant-based foods 15% (men) to 18% (women). With respect to the contribution of different food groups in Iceland, the consumption of beef and lamb (49 g/day) contributed 44% of total GHGEs, whilst the consumption of seafood (45 g/day) and pork and poultry (49 g/day) contributed 20%. Those with high adherence to plant-based or vegan diets had considerably lower GHGE compared to those not adhering to such diets (mean: 2.6 vs. 6.1 kg CO_2_-eq./day), whilst the difference was somewhat smaller when standardised to 10 MJ (4.0 vs. 6.9 kg CO_2_-eq) (26). Another study found the share of GHGE from beef (52 g/10 MJ, raw and processed weight) in an average Danish diet of 18%, when applying the country-specific process-based Life Cycle Assessment (LCA) data (attributional-LCA, a-LCA), but 45% when applying data using top-down input–output analysis, an economic method assuming a direct correlation between cost and environmental impact (28). The database relying on the top-down analysis, which was also applied for the Icelandic calculations, has different GHGE values than the a-LCA-data, for example, higher for beef and lamb but lower for milk and several plant foods (28). In a Finnish study, 12% of GHGE of total food purchases was from beef (35).

Discretionary foods, such as sugar, sweets, snacks and beverages, including coffee, tea and alcoholic and sweetened drinks, also contribute to around one-fifth of dietary GHGE in studies of Nordic diets (19, 18 and 22%, respectively), which may reflect the high consumption of such foods (28, 30, 38).

Women’s food consumption has had lower GHGE than men’s in several Nordic studies (24, 28, 31, 37, 39, 40). In some studies, no gender difference was present when adjusted for energy intake (28, 31, 37), whereas in a Swedish cohort of adults, women had 20% lower GHGE than men for the same energy content for both women and men) (39). Also, amongst Swedish adolescents, GHGEs were higher in men than women (medians 4.2 vs. 3.8 kg CO_2_e/10 MJ) based on data from the national dietary survey ‘Rigsmaten 2016–2017’ (41). In women, the proportion of GHGE from animal-based foods was lowest and plant-based foods and sweet foods/beverages was highest in the highest grade (grade 11, aged 17–18 years). In males, these proportions were similar across grades. Different measures of nutritional or health quality showed different relations to CO_2_e. The Nutrient Rich Food Index (NRF11.3) was not associated with CO_2_e/10MJ, whereas healthier eating, according to Swedish Healthy Eating Index for Adults 2015 (SHEIA15), was inversely associated with CO2e/10 MJ (41). In Sweden and Finland, dairy had the largest climate impact amongst women and red meat amongst men (24, 31, 36).

In the study by Mertens et al., both GHGE and land use increased with increasing energy intake and also increased with age in Danish individuals (participants aged 18–64 years) (37). The higher GHGE in older individuals (50–64 years) was explained by higher consumption of meat, fish and eggs, whereas higher land use of older individuals was explained by higher consumption of beverages (including coffee, tea and alcoholic and sweet beverages) (37). In a Swedish population aged 29–65 years (mainly between 40 and 60 years), younger age, higher BMI, higher education level and living in urban area were associated with higher GHGE (42). Also, amongst Swedish adolescents, overweight/obesity was positively associated with CO_2_e/10 MJ (41).

Whilst animal-sourced foods significantly contribute to many of the environmental impacts, in the study by Hallström et al. on Swedish diets, discretionary foods and plant-sourced foods also make notable contributions to consumptive water use and extinction rate (31). Vegetables, fruits and berries, and nuts and seeds were responsible for 33% of consumptive water use in men and 48% in women, and 29% of extinction rate in men and 43% in women. Discretionary foods were responsible for 41% of consumptive water use in men and 33% in women, and 37% of extinction rate in men and 33% in women. Discretionary foods also had a substantial contribution (30%) to the dietary impact on phosphorus application. The food group with the largest impacts within discretionary foods was primarily coffee (31). In a Finnish study on land-use-related biodiversity impacts of the current average Finnish diet [based on the current diet in Saarinen et al. (25)], meat emerged as the primary contributor to the biodiversity impact, with poultry contributing the most and beef the least (43). Coffee and dairy also contributed significantly. Biodiversity impact of land use change was strongly associated with meat consumption, whereas land occupation was associated especially with beverages, sugars and sweets (43). Studies on water use as part of Nordic food consumption suggest that a large share of the water footprint of the current diets, measured as scarcity-weighted or blue water use, derives from foods such as citrus fruits and rice (44, 45), and in Denmark also from wine and olive oil (44). The water use occurs almost entirely (>90%) outside the Nordic countries due to the imported foods (44, 45). The crops grown in the Nordic countries contribute only little because of abundant water resources in the region (i.e., water scarcity is rare) and because the weather conditions most years allow rainfed farming instead of irrigated farming (46, 47).

Studies have also shown that food losses and waste along the food chain (24, 25, 28, 48, 49) contribute to increased environmental impact: food losses and waste at the consumer level accounted for up to 18% of total GHGE of the diet. In addition, excessive food intake also contributes to increased environmental impact from food consumption, which in a Swedish study amounted up to 10% of the total food-related GHGE (50–52).

### Environmental impacts of dietary change in Nordics. Scenarios and modelling studies

#### Pre-defined scenarios adhering to FBDG

Nordic studies (Table A1, Appendix A) show that switching from the current Nordic diets to the diets adherent to the current national FBDG and/or meeting nutrient recommendations would reduce GHGE by 8–45%. In a Finnish study, GHGE could be reduced by an average of 27% for men and 15% for women if diets were changed to comply with the current recommendations for nutrient intake (40). Trolle et al. studied the change in GHGE when transitioning from the current Danish diet to a plant-rich diet, with equivalent energy intake, and demonstrated a 31% reduction in GHGE when applying attributional LCA data and 45% reduction when applying top-down consequential LCA data (28). The plant-rich diet was developed by simple modelling of the global reference diet from the EAT-Lancet Commission into a Danish context. The modelling considered the prevailing food culture and local food availability (e.g. including only a few fortified products), and nutritionally adequacy within the age range of 6–65 years, and the latest evidence on food intake and risk of chronic diseases (53). Compared with the current diet, the total amount (in grams) of meat in the plant-rich diet was about one-third, and the amounts of fish and plant foods were increased (Table 1) (28, 53). Saxe et al. compared GHGE of production of a diet adherent to the NNR2004 (NNRD) and the New Nordic Diet (NND), which is based on locally and mainly (75%) organically produced Nordic foods. NNRD and NND include less meat and discretionary foods and beverages, and more dairy products, fruits and vegetables than the average Danish diet (survey data before 2006) (38). The GHGE from the NNRD and the NND was 8 and 7%, respectively, lower than the average Danish diet, with all diets adjusted to equivalent energy content. In a Swedish study (54), GHGE was reduced by roughly 20% if the consumption followed the Swedish FBDG 2015 [based on NNR2012 (6)], calculated with a simplified diet where each food group was represented by a few food items. Acidification was reduced by one-third, eutrophication by ~30%, land use by ~20%, terrestrial ecosystem toxicity by ~5%, human toxicity by ~8% and biodiversity damage potential by ~25%. The modelled diets were neither tested for nutritional adequacy nor adjusted for energy content (54). Also, the modelling illustrated that, in general, organic foods do not have a lower impact on GHGE. The scenario with 100% organically produced foods, however, showed reductions in ecotoxicity (−60%), human toxicity (−35%) and biodiversity damage potential (−30%). Finally, a scenario with a 30% increase in foods produced in Sweden showed equal or minimally reduced environmental impact in all impact categories, compared with the current Swedish diet (54). A recent Norwegian study by Wright et al. (55) showed changes across six environmental impact categories when comparing the current diet amongst 2-year-olds to two scenario diets based on the Norwegian FBDG and the more plant-based EAT-Lancet reference diet, with the energy intake of 5.3 MJ/day corresponding to the reference energy intake for 2–5 years-old (NNR2012). Compared with the current diet, the FBDG scenario demonstrated minor to moderate reductions: 7% for global warming potential (GWP), 3% for land use, 2% for freshwater eutrophication, 8% for marine eutrophication, 35% for water use and 18% for terrestrial acidification. The corresponding impacts for the EAT-Lancet scenario diet exhibited even more substantial declines, with reductions of 37, 7, 38, 5, 56 and 59%, respectively (55). Milk and dairy products emerged as the main contributors to environmental impacts in both the current diet and the FBDG scenario diet. The scenario diets were nutritionally adequate and improved the dietary quality amongst Norwegian 2-year-olds (55). In nine Nordic cities, the freshwater resources of modelled diets constructed to follow the NNR2012 led to a lower water footprint than the current diet in each city (diets equivalent by energy and protein content) (56). The differences were mainly attributable to reductions in meat and dairy products. A global comparison by Springmann et al. [with data sources described by Harwatt et al. (9)] demonstrated that adhering to the national FBDG from 2012 to 2018, alongside a shift from the current energy intake to recommended levels, would yield the most substantial modelled reduction in GHGE in Iceland (−32%) (33).

**Table 1:** Overview of the current FBDG in the Nordics, with amounts provided for specific food groups

Food group	Denmark 2021	Finland 2014	Iceland 2014+later amendments	Norway 2014	Sweden 2015
**Vegetables, fruit, and berries /day**	□ 600 g	□ 500 g	>500 g	□ 500 g	Eat a lot 500 g
**Potatoes**/day	mentioned as part of a healthy diet 100 g suggested	recommended to keep at current intake level	mentioned as part of a healthy diet	mentioned as part of a healthy diet	Mentioned as part of a healthy diet
**Pulses (legumes)**	100 g/day (cooked)	included in vegetables	included in vegetables	encourage to eat	included in vegetables
**Nuts (and seeds) /day**	30 g +1–2 tablespoons seeds	30 g (2 tablespoons) unflavoured	30 g	not included, encourage to eat one handful unsalted	2 tablespoons
**Whole grain /day**	At least 75 g	At least 3 (women) to 4.5 (men) portions	At least 2 portions (70 g)	70–90 g	70–90 g
**Fish and seafood/week**, cooked weight	350 g (200 g fatty fish)	2–3 times (200–450 g)	2–3 times, includes fatty fish once a week in all groups, corresponding to 300–450 g	300–450 g (200 g fatty fish)	2–3 times
**Meat and meat products/week, cooked weight**	Max 350 g (all meat)	Max 500 g of red and processed meat, corresponding to 700–750 g of raw meat	<500 g red meat and processed red meat	Max 500 g red meat and processed red meat	Max 500 g red meat and processed red meat
**Milk and dairy products/day**	250 mL (low fat) and 20 g cheese	5–6 dL of liquid milk products (max 1% of fat) and 2–3 slices of cheese (max 17% of fat)	2 portions or 500 g low fat and without added sugar	3 portions (low fat), e.g., 4 dL milk and 20 g cheese	2–5 dL
**Fats and oils**	Vegetable oils rather than hard fats such as butter and coconut oil	Choose plant-based margarines (min 60% of fat) and vegetable oils	<10 E% SFA Vegetable oils rather than hard fats such as butter, margarine, and coconut oil	Choose margarine and oils, not butter	Switch to healthy fats!
**Beverages**	water	Preferably water	water	water	
**Sugar-containing foods and drinks**	Limit intake. Max intake defined age/gender groups	Not regularly	<10 E% from added sugar. Limit intake of sweets	Limit intake. <10 E% from sugar	Limit intake

DK Fruits+veg: at least half veg, choose high-fibre veg, dark green veg and red and orange veg. Berries included in fruits.

Fin Fruits+veg: 5–6 portions per day (about one-half berries and fruits and one-half vegetables). Portion: a medium-sized piece of fruit, 100 mL of berries or 150 mL of salad or grated vegetables.

DK whole grain, preferably more.

Fin cereals: Six portions for women and nine for men daily with at least one-half whole grain. One portion refers to 100 mL of cooked whole-grain pasta, barley, rice or other grain side dish or one slice of bread.

#### Meat and fish scenarios

A reduction in meat consumption is associated with reductions in environmental impacts, as shown in scenarios based on Nordic diets (25, 28, 40, 49, 54, 57, 58). A Finnish study found a decrease in GHGE by 50 and 60%, respectively, when either one-third or two-thirds of the meat and dairy was substituted with legumes and plant-based dairy alternatives (40). In these scenarios, energy intake was held constant, whilst the consumption increased to the recommended levels for fruit and vegetables (≥500 g/day) and fish (2–3 servings/day) (40). In addition, these modelled diets were combined with strong national restrictions to reduce the cultivation of peat fields, the main contributor to GHGE in the Finnish food system (59). Without peat field changes, the GHGE reductions would be only 10%. In a Swedish study, Röös et al. explored a scenario in which meat consumption was reduced by 50% and replaced with similar edible weight of domestically grown grain legumes (55 g/day, cooked weight) (58). The transition would maintain nutrient intakes within the NNR and greatly improve intakes of fibre and folate. The climate impact (−20%) and land use (−23%) associated with the Swedish diet as well as the need for nitrogen fertiliser and the nitrogen load from wastewater plants would be reduced (58).

In a Finnish dietary scenario (based on Saarinen et al.: ‘meat to half of the current diet’, ‘meat to a third of the current diet’, ‘fish and milk rich diet’ and ‘a vegan diet’), land use-related biodiversity impact decreased along with a concurrent decrease in animal-sourced food content of the diet (43).

#### Vegetarian and vegan scenarios

Vegetarian and especially vegan diets have consistently been shown to cause the least climate impact in the Nordic modelling studies (25, 29, 33, 54, 60) (Table 1). Bruno et al. investigated the GHGE of carnivore, vegetarian and vegan diet scenarios with equivalent energy intakes, where animal-based products were replaced with pulses, beans, peas and nuts (29). The GHGEs were largest for the carnivore diet (1.83 t CO_2_-eq./capita/y), followed by the vegetarian (1.37) and vegan (0.89) diets. The primary production phase was the most important contributor to the GHGE values of foods (65–85%). The study did not consider nutrient content of the diets (29). Saarinen et al. modelled that the largest reduction of GHGE (37%) was a result of shifting from the current average diet to a vegan diet, although the energy content of the modelled vegan diet was over 500 kcal larger than the current diet (1,949 kcal/d) (25). The reduction in GHGE was even larger when the energy intake was equivalent to the current diet (52%). However, the vegan diet deviated from several nutrient recommendations already in the higher energy intake scenario (e.g. was too low in selenium and iodine), suggesting even larger deviations in a scenario with energy intake similar to the current diet (25).

In a Swedish study, the reduction in GHGE was even larger, roughly 40 and 70% relative to the current average Swedish diet of a vegetarian and vegan diet, respectively (54). The nutritional content of the diets was not assessed. In addition to reducing GHGE and acid emissions, vegetarian options (grain products, potatoes and vegetables) also reduced the total environmental burden: land use, acid emissions, fuel and electricity consumption, the total material requirement of the diets (60) and resulted in the largest reductions in the water (blue and green) footprint (56).

Springmann et al. demonstrated that in the Nordic countries, the maximum GHG reductions would be 85% for shifting to pesco-vegetarian, 85% for vegetarian and 91% for vegan diet with equivalent energy content between the diets (61). In the vegetarian or vegan scenarios, riboflavin remained low, and calcium and vitamin B12 fell below recommended intakes (61).

In a Finnish study, land use-related biodiversity impact of a vegan diet was 30% of the impact of the current average Finnish diet (43). Within the vegan diet, beverages, legumes and nuts made the greatest contribution to the reduction in biodiversity impact.

#### Optimisation studies

Whilst nutritionally adequate, dietary patterns have been built by simple iterative modelling (53, 62), also mathematical methods have been applied for developing optimised diets that are both nutritionally and environmentally sustainable by imposing different constraints and objective functions (63, 64). Optimisation studies on the Nordic diets have arrived at conclusions in line with above-mentioned studies on existing dietary patterns and scenario studies.

Using data from five European countries, including the national dietary surveys from Finland and Sweden, Vieux et al. investigated the dietary changes needed to reduce the GHGE of diets, whilst maintaining nutritional adequacy and constant energy intake. Diets following a stepwise reduction of the carbon footprint (10% steps) were modelled using linear programming (65). Depending on country and gender, maximal GHGE reductions are achievable whilst still ensuring nutritional adequacy ranged from 62 to 78%. Along with reductions in animal-based food consumption, a substantially increased contribution to energy from fruits, vegetables, legumes and starchy foods and a decreased contribution of energy from sugar and fats were needed (65).

Using linear programming optimisations, Mazac et al. modelled sustainable European diets using the EFSA Comprehensive Food Consumption Database from 22 European Union countries and the LCA inventory data for food from the French database AGRIBALYSE, and for novel/future foods from recently published literature (66). The aim was to optimise diets that meet the recommended daily nutrient intakes, assuming unchanged energy intake, as well as to minimise environmental impacts (GHGE, arable land use as m^2^ eq., and consumptive water use, that is, consumed water that will not return to the watershed). All impact categories were minimised to produce three separate diets with differing constraints: a diet without animal-based foods, a diet with animal-based foods available for optimisation and a diet with novel/future foods, such as cultured meat and ovalbumin, whilst excluding animal-based foods. The food composition of the diets was different depending on which of the environmental impacts were minimised, but all deviated markedly from the current diet. For the diet with animal-based foods as an option, the optimisation model did select dairy in the land use and water use minimisation models but not in the GHGE minimisation model. Instead, the meat was primarily replaced with fortified liquid plant-based products such as plant-based dairy alternatives, grains, fruits, vegetables and tubers, and a small amount of legumes. All environmental impacts decreased (up to 80%) with and without novel foods. In all modelled diets, large increases in fortified liquid plant-based products were needed to meet calcium and vitamin D requirements (66). Without supplements, the vegan diet was inadequate in vitamins D and B12.

Using a mathematical benchmark optimisation method for diets in four European countries, including Denmark, Mertens et al. modelled diets with equivalent energy intakes and improved nutrient quality and climate comprising of existing dietary practices (67). When climate impact was prioritised, a nutrient index (Nutrient-rich diet [NRD] 15.3) was ~9% higher and GHGE ~21% lower and ~73% of food intake remained similar to the observed diet. This required consumption of red and processed meat to be partly shifted to either eggs, poultry, fish or dairy. The diet with the highest nutrient quality did not result in a lower GHGE, whereas the diet with the largest improvement in GHGE led to a higher nutrient quality but did not achieve the full health potential in terms of nutrient content (67). When red and processed meat in the previous scenarios were substituted by similar energy content from meat replacers (e.g. Quorn, vegetarian burgers, falafel and meat analogues), the GHGE reduction was 24–39%, and on average, an additional 3% with theoretical fortified meat replacers (68). As a recent Danish study using quadratic programming to minimise the departure from the average diet as a proxy for acceptability or feasibility of dietary changes, Nordman et al. found GHGE of the optimised diets as follows: 3.93 kg CO2-eq (with only nutrient constraints), 3.77 kg CO2-eq (with nutrient and health-based food group constraints) and 3.01 kg CO2-eq (with nutrient, health and GHGE constraints), in contrast to 4.37 kg CO2-eq in the observed diet (28, 53). Compared with a plant-rich diet aligned with the official Danish FBDG (28, 53), the optimised diet included, for example, more pork and less legumes, but the climate impact was similar. The optimised diet’s food composition deviated on average less from the observed diet than the Danish plant-rich diet, potentially being more acceptable to some individuals. However, the optimisation focusing solely on GHGE may not yield a diet that is more sustainable across other environmental aspects (69).

Applying Finnish data, Valsta et al. (40) performed mathematical optimisation to model nutritionally optimal diets with 33 or 50% reduction in GHGE. In the models, the deviations of the food intakes from the current food intake were restricted, energy content held constant, but adhering to FBDG was not required. Both levels of reductions of GHGE suggest replacing meat and dairy consumption with mainly vegetables, fruits, legumes, grains and potato. Fish consumption in the model increased in women but decreased in men. Egg consumption remained almost unchanged in the 50% GHGE reduction model (24 g/day), but in the 30% GHGE reduction model, egg consumption increased in women but decreased in men. Jalava et al. modelled diets with stepwise reductions in animal-protein intake and optimised these to meet the energy and nutrient recommendations and to minimise changes in the food amounts (70). The results for Europe, excluding East European countries, show that, compared with the current diet, the diet following dietary recommendations (with max. 200 kcal lower energy content than the current diet) reduced the total water use (blue and green) by 13%, whilst the diet without meat decreased the total water footprint by 30% (70).

An optimisation by linear programming was done based on foods and drinks supplied to Swedish schools (71, 72). In a holistic approach, GHGE was first mathematically minimised, whilst simultaneously integrating aspects of health, affordability and acceptability, and keeping energy content constant. Models were all nutritionally adequate but differed in GHGE. Pulses, cereals and eggs increased, and fats and oils, and dairy decreased. The amount of ruminant meat decreased in favour of other meat products, whilst the total amount of meat was practically unchanged. The authors concluded that moderate changes were needed to achieve lower GHGE from the food supply to school lunches that were omnivorous, nutritionally adequate and affordable (71).

#### Intervention studies

According to a Finnish 12-week randomised controlled trial (RCT) using a whole-diet approach, the partial replacement of animal-sourced proteins with plant-sourced proteins in iso-caloric diets affects the climate impact (GHGE), and nutrition and health outcomes of the adult Finnish diet (73–76) reduction in GHGE were observed when the ratio of animal to plant protein sources was altered from the Finnish average of 70:30 to 50:50 (−20% CO_2_-eq./d) and further reduced to 30:70 (−39% CO_2_-eq./d) (76). The reduction in GHGE was considered significant, given that the change in diet could be relatively easily implemented. There were several health-promoting dietary changes such as increased fibre intake and improved dietary fat quality and blood lipoprotein profile (73). However, increased markers of bone resorption and formation were observed, which indicates a possible risk for bone health (74). Markedly reduced intake and status of vitamin B12 and iodine were also seen after the intervention (75). It is noteworthy that the participants were allowed to use neither dietary supplements nor the foods and drinks fortified with the assessed nutrients, except for iodised salt as an ingredient in cooking and bakery and industrial products.

In a system-level pilot study by Elinder et al. (72) and an intervention study by Colombo et al. (77), the effects of implementing optimised lunch menus on food waste, consumption and pupils’ school meal satisfaction were assessed in Swedish primary schools. The lunches were optimised to minimise deviance from the current lunches. Meeting nutritional recommendations for school lunches and GHGE at a maximum of 500 g CO2 eq./lunch was set as a constraint. The optimised solution with unchanged energy content included fewer dairy products more pulses and cereals, and slightly more egg. Ruminant meat was substituted with lower GHGE meat, such as poultry, with the overall amount of meat remaining unchanged. The food lists developed based on the results of the optimisation were 28% (72) and 40% (77) lower in GHG, met all nutrient recommendations for school meals and cost less than the baseline lists. Plate waste, serving waste, consumption and school lunch satisfaction did not differ significantly from the baseline diets.

## Environmental sustainability in FBDG of the Nordics

Sweden provided a scientific basis for environmental assessment of the national FBDG (4). In addition to several international and Nordic scientific reports and papers, the Swedish report [and a Danish report (78, 79)] formed the basis for the section on sustainable food consumption in the NNR2012 report (6) (Nordic).

In the NNR2012, FBDG focused on plant-based foods. Environmental sustainability was based on a narrative review of the literature. It provided a detailed overview including introduction to planetary boundaries, addressing the toxic impact, biodiversity, eutrophication, acidification, land use, land use change and water use, but focusing on GHGE and the need for reduction to reach the EU goal of GHGE reduction by 2050 compared with the 1990 level. It also addressed possible dietary changes from the present diet to an environmentally sustainable diet, indicating positive and negative health and environmental effects for each food group. The dietary changes were expressed as ‘less of’ ruminant meat, e.g., pork and poultry, dairy milk, cheese, butter, palm oil, savoury snacks and sweets, and ‘more of’ fish and seafood (wild and farmed), e.g., eggs, fruits and berries, field vegetables, green house grown vegetables (less fossil fuelled), potatoes, legumes, nuts and seeds, cereals and grains, vegetable oils and water as drink. Also, changes to more organic food were included with less or no pesticide use as positive environmental effect but a comment of ‘lower production per hectare’. Several concerns were expressed, for example, about overexploitation of fish populations and risks of pollution from farmed fish. It was concluded that the uncertainties in calculations were large, and in spite of possible conflicts exist between nutritionally and environmentally sustainable diets, it was concluded that there are promising possibilities to eat nutritionally adequate and varied diets in a sustainable way (6).

The degree to which environmental sustainability is included in the current national FBDG differs between Nordic countries. The ‘Norwegian guidelines on diet, nutrition, and physical activity’ (80) are health-based, based on a systematic review of scientific evidence. Emphasis is placed on results from epidemiological studies, biological/mechanistic studies and clinical trials. Only when the overall evidence is characterised as convincing or probable, is it used as a basis for dietary advice (81). The Norwegian FBDG was assessed in a sustainability perspective by a working group assigned by the Norwegian Nutrition Council (82), and supplementary advice for each advice in the guidelines was suggested to the authorities.

In Finland, the FBDG (83) is mainly based on NNR2012. In the Finnish FBDG, the environmental sustainability perspective is included as a separate section in all current nutritional recommendations.

The Icelandic FBDG is health-based and encourages individuals and families to reduce food waste by organising food purchases and cooking to maintain a healthy diet and minimise food waste, thereby supporting the environment (84). Furthermore, the general Icelandic FBDG explains how increased consumption of plant-based foods and decreased consumption of animal products will lead to less GHGE and protect the environment.

The Swedish FBDG (85) is health-based, and information on the environmental impact of each food group is based on NNR2012 and provided in close connection to the FBDG. The development of the guidelines was carried out in collaboration with different stakeholders through a reference group with representatives from other governmental bodies and research centres. An open hearing was conducted, and the guidelines were piloted with consumers to ensure comprehension in terms of message content, language and choice of images.

**Fig. 1:**
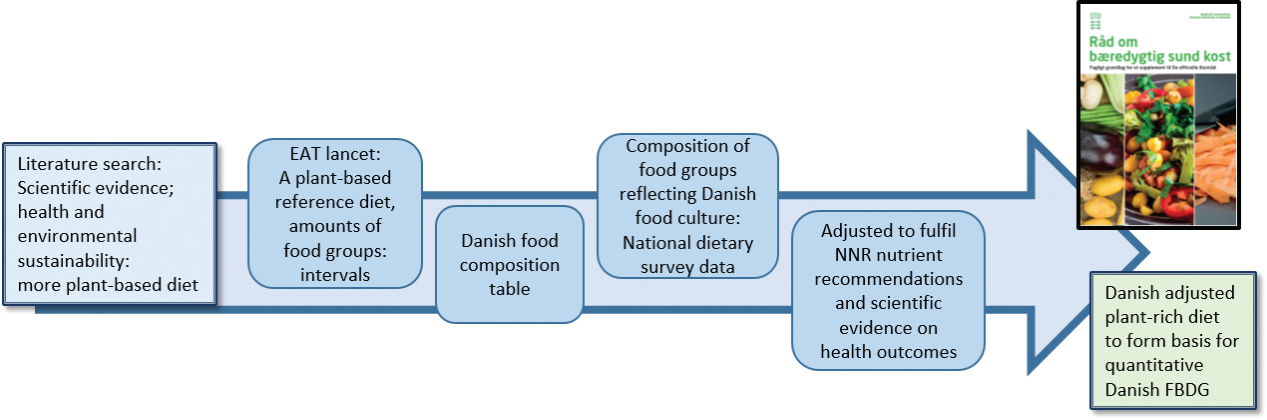
Modelling process of developing the Danish plant-rich diet underlying the official Danish FBDG (provided by the team in (53)).

The most recent official Danish FBDG (‘good for health and climate’) (86) is based on both health-based evidence for associations between intake of foods and risk of disease and published literature for environmental footprints of foods and dietary patterns and dietary modelling to ensure nutrient adequacy (53, 87) (Fig. 1). Danish Food and Veterinary Administration chaired the development process for deciding how to communicate the FBDG, which involved workshops and dialogue with a broad range of stakeholders, and a pre-test of understanding and interpretation of the dietary guidelines amongst Danish consumers.

### Comparison of FBDG of the Nordics

In all Nordic countries, the FBDG consists of 7–12 main dietary messages (see Appendix C), which are explained and quantified in supplementary texts. All mention variation, either as an overall principle or within specific food groups such as vegetables, fruits and berries, and fish. Physical activity is promoted in the heading or mentioned as a basis for the FBDG. Also, the FBDG includes advice on not overeating and limiting or reducing food waste. In addition, choosing products with the keyhole label (Denmark, Iceland, Norway and Sweden) or the heart symbol (Finland) is recommended. The FBDG of the Nordics provides advice regarding the different food groups included in Table 1.

All countries promote eating more fruits, berries and, especially, vegetables. The amount for all countries is 500 g/day (excluding potatoes), except for Denmark (600 g/day). The supplementary advice varies. Denmark emphasises dietary fibre-containing vegetables, dark green vegetables and red/orange vegetables, which are important in the plant-rich diet with a low content of meat. All countries promote vegetable oils, potatoes (except Sweden) and whole grains, although with slightly different means and amounts. In Finland, at least half of the recommended daily consumption of cereal products (three of six servings for women and about 4.5 of nine servings for men) is to be whole grains. The fibre content in bread should be at least 6 g/100 g. All FBDGs include around 30 g of nuts. All countries also promote fish and seafood, for example, as 2–3 times a week, Iceland and Norway with a target amount of 300–450 g cooked weight/week, Finland (200–450 g/week) and Denmark 350 g cooked weight/week. All FBDGs include advice on reducing discretionary foods (sugar-rich, high-density foods, sweet beverages, alcoholic drinks, chips and other snack products), salt and saturated fat.

The main differences between the Nordic countries are seen for meat, milk and pulses (or legumes). Regarding meat, in Denmark, the focus is on total meat (‘Eat less meat – choose legumes and fish’) with a recommended maximum total meat quantity of 350 g cooked weight/week, and reducing the intake of red meat, particularly ruminant meat and processed meat. The reduction of red and processed meat is health-based, and the reduction of total meat would also reduce the environmental footprint. The other countries focus on red and processed meat, mainly based on health evidence, with a maximum of 500 g cooked weight/week, and give additional advice on reducing the intake of ruminant meat, and in Sweden also on other types of meat. Supplementary advice encourages a preference for low-fat and low-salt meat products.

All FBDGs include low-fat varieties of milk and dairy, but the definitions and the amounts differ from 2.5 dL liquid dairy + 20 g of cheese in Denmark to 5–6 dL + 2–3 slices of cheese per day in Finland. Finally, all FBDGs encourage the consumption of pulses (or legumes), and only Denmark provides a target amount of 100 g/day (cooked) of pulses – defined as dried or ripened beans, peas and lentils – not including fresh green peas and green beans.

### FBDG for specific population groups and professional kitchens

All the Nordic countries have published additional FBDG for various population groups with specific nutrition needs, for example, infants and children, and pregnant and lactating women. Guidelines for professional kitchens, for example, in geriatric homes, early childhood education and care and schools are also published. Moreover, general guidelines have been issued for vegetarians and vegans in Norway and Sweden, for young vegan children and pregnant and lactating vegan women in Iceland, and for vegetarians in Denmark (see references in Appendix C).

### Approaches for developing FBDGs that include environmental sustainability

With respect to the UN Sustainable Development goals (88) and the FAO/WHO guiding principles for Sustainable Healthy Diets (7), several studies have investigated methodologies to assess sustainability characteristics of diets when developing FBDG (62, 89–92). Bechthold et al. identified eight aspects: diet-health relations, nutrient supply, energy supply, dietary habits, environmental sustainability, food-borne contaminants, target group segmentation and individualisation. They concluded that the first four aspects had already been widely applied in existing FBDG; the others had almost never been considered (89). The study by Mazac et al. draws similar conclusions, using a qualitative content analysis in a framework with five interconnected core domains: health and nutrition, food security and agriculture, market and value chains, sociocultural and political, and environment and ecosystems. As the method mainly helps to validate previous work, further development is needed to improve the guidance approach for the incorporation of sustainability domains in future FBDG (91).

Based on studies, aiming at helping interpretation of studies and researchers in choosing the most relevant methodology for future studies, four development approaches emerged: 1) hypothetical diets or 2) observed diets, 3) identifying more sustainable diets than others from observed diets or 4) designing sustainable diets using mathematical optimisation (92). Data requirements vary between the four approaches: individual-level or average food consumption data. All approaches could use preferably one or more of environmental impacts, for example, GHGE, land use, water use, N and P use and impact of human toxicity and biodiversity, as well as evaluating nutrient adequacy and adhering to health-based evidence of consumption of foods, divided into food groups. The ability to capture perspectives of acceptability/dietary preferences and variability in impact between population groups varies between the approaches (92).

In Denmark, a combination of Approach 1, Approach 2 and simple optimising modelling of average food consumption was used to ensure nutrient adequacy. It resulted in a plant-rich diet low in meat, which formed the basis for revised FBDG in Denmark (53), as illustrated in Fig. 1.

The health evidence was mainly based on a Danish review (93), which drew on the comprehensive work from Norway (81), and was supported by newer systematic reviews. The environmental sustainability evidence was based on a narrative literature review (87). For both health and environmental sustainability, it was concluded that the diet should change towards a more plant-based diet. The suggested ranges for selected food groups based on the health evidence and additional ranges from the global plant-rich reference diet by the EAT-Lancet Commission (32) formed the basis for the principles of the iterative modelling of the average food consumption data from the National survey on dietary habits and physical activity. The nutrient recommendations of NNR2012 and the Danish food composition data were also applied and resulted in a plant-rich, nutritionally adequate diet (53) (Fig. 1). Similarly, adequacy of the plant rich diet for 2–5 years, elderly aged of 65 years and above, and pregnant and breastfeeding women was ensured, and specific guidelines were included in the FBDG for these groups.

Wood et al. have described a framework for developing FBDG that meet health and nutritional goals whilst simultaneously meeting quantitative environmental sustainability targets. The framework is science based, building on Nordic experiences, and provides feasible and flexible solutions when piloting it in the Swedish context (62). Five steps are included in the framework:

*Step 1: Determine an average healthy diet for a given population and criteria for healthy diets.*

*Step 2: Identify relevant environmental aspects and establish corresponding boundaries.*

*Step 3: Identify systemic effects and crucial sustainability aspects.*

*Step 4: Alter the average diet to meet environmental goals and resolve trade-offs between environmental and nutrition goals.*

*Step 5: Formulate sustainable food-based dietary guidelines.*

Step 1 includes identifying an average healthy diet by establishing criteria for healthy diet and ensuing the average healthy diet meets the criteria. In a Nordic context, this step could build on the health-based FBDG established in the Nordic countries in combination with the updated work of the NNR2023 revision on health-based FBDG and nutrient DRVs.

In Step 2, this paper illustrates the use of the framework by adopting the environmental aspects used by the EAT-*Lancet* Commission. However, the authors note that different environmental aspects, such as water scarcity or local biodiversity impacts, could be included to better represent national environmental concerns. Step 2 includes establishing environmental boundaries, establishing a target year and determining the mitigation potential from waste reductions and production improvements. To illustrate how boundaries for the average Swede could be determined, Wood et al. downscaled the Eat-Lancet boundaries to per capita allocations. However, for other sharing principles used to allocate impacts, this paper refers to Ryberg et al. (94). Wood et al. use 2030 as the target year, diverging from the EAT-*Lancet* food system targets year of 2050 (32). Wood et al. assume that the environmental impacts of diets reach halfway between the current impact and the EAT-Lancet target by 2030. They adapt the SDG [UN Sustainable Developmental Goals (United Nations 2015)] for waste reduction by 50% by 2030 and assume that half of the production improvements forecasted by 2050 can be met by 2030. It was emphasised that these decisions and assumptions were examples, and any national development process using this framework should include relevant stakeholders and expertise. Stakeholder engagement is also crucial for Step 3 *identify systemic effects and crucial sustainability aspects.* These aspects can vary widely based on the context but can include coupling ratios, such as the meat to offal ratio, dairy to beef ratio and dairy to dairy fat ratio. Aspects can also include country-specific issues such as goals for organic food production and consumption and the local biodiversity impact of grazing by ruminants, which, to a certain extent of grazing, might improve biodiversity whilst overgrazing might be harmful. The authors stress that the aspects identified in Step 3 cannot be used as a justification to relax environmental boundaries already established in Step 2. In Step 4, the environmental performance of the diet is assessed. The key question here is what data to be used, and this paper provides comprehensive considerations on this. The step includes an iterative modelling process where changes to the Step 1 healthy diet aim to reduce environmental impacts of diets within the Step 2 boundaries, whilst maintaining nutritional adequacy in relation to the established nutrition targets and food system boundaries. Trade-offs between nutrition and environmental goals are considered in this step, and the authors suggest that a deliberative process with stakeholders can be used to resolve these trade-offs. Steps 1–4 result in a healthy and environmentally sustainable diet that can form the basis for communicating of both qualitative and quantitative SFBDG in Step 5, where stakeholder consultation is recommended. The paper by Wood et al. presents the food composition for one example of a healthy and environmentally sustainable diet that is adapted to the Swedish population (62). The food composition of this diet is close to the plant-rich diet underlying the Danish FBDG (2021), although with higher content of total meat (pork and poultry) and less pulses. The authors (62) illustrate that this example diet meets the example nutritional criteria set out in Step 1 whilst also being within the example environmental boundaries established in Step 2 for climate change, land use change, nitrogen cycling, phosphorous cycling, freshwater use and biodiversity loss.

An advantage of the framework is its systemic approach, which permits inclusion of considerations inherent to food systems, that is, animal welfare considerations. The authors suggest that considerations directly related to dietary patterns should be included, whilst indirectly related sustainability aspects, such as labour conditions or market access issues, and animal welfare, are handled more efficiently in other policy processes. Lack of data and data quality may be limitations in the use of the framework, yet the authors discuss alternative methods and data sources for the steps.

## Discussion

To summarise, Nordic studies reviewed for this paper demonstrate that the current diets are far from environmentally sustainable, indicating that food consumption in the Nordics exceed planetary boundaries for most impact categories. A fundamental question revolves around how to partially replace animal-sourced foods, namely, meat and dairy, to develop diets that meet both nutritional and environmental objectives. Transitioning towards more plant-based diets can have both positive and negative nutritional effects depending on dietary composition. However, the overall dietary quality would most often improve with improved health outcomes due to higher intake of vegetables, fruits, whole grains and legumes and lower intake of red and processed meat and discretionary foods, which is confirmed in other international studies, such as the Global burden of diseases (95, 96, 12).

From different observed dietary patterns in Nordic countries, more plant-rich patterns consistently showed lower GHGE and land use than current diets with high content of meat, and high GHGE, in particular from ruminant meat, and dairy products. Due to high consumption, discretionary foods including coffee, tea and alcoholic beverages make a substantial contribution to the environmental impacts of the current diets. Most commonly, the reviewed studies reported only GHGE, but studies that include more environmental impacts show that GHGE alone does not encompass the full range of environmental impacts associated with diets.

The finding that the current diets exceed the planetary boundaries for GHGE, cropland use, nitrogen and phosphorus application, and biodiversity (30, 31, 62) emphasises the need for urgent changes in the Nordic food system. The studies also show that water use from the current Swedish diet does not exceed the planetary boundary, but since water use is a larger issue for imported foods, some foods come from water scarce areas; changes in the water resources may occur along with climate changes, and water use must not be neglected.

Animal-based foods, especially meat and dairy products, were the largest contributors to dietary GHGE and land use. Country- and gender-specific differences were apparent. The gender differences stem mainly from the higher energy intake of men relative to women, but also from differences in diet composition between the sexes. For example, in Sweden and Finland, the largest contributor to the carbon footprint in the diet of men was meat, whereas the largest contributor in the diet of women was dairy (24, 36). Marked variation in estimated GHGE and land use was found in diets between Nordic countries and within countries. This may be partly attributable to different data and methodological choices in studies as indicated in Appendix B, but results also suggest that cultural differences in food composition of diets may be an important factor when establishing and implementing national FBDG.

The results of the Nordic modelling studies confirm the potential of shifting towards a plant-based diet in reducing GHGE, also decreasing land use, acid emissions, fuel and electricity consumption, and total material requirement of the diets. Our findings showed a reduction in GHGE, up to 80% (when novel and fortified foods were included in the optimised models) but typically lower in other models. These results align with a recent study comparing impacts of different self-selected dietary patterns (97) and with several reviews, for example, Aleksandrowicz et al. (98) showing reductions above 70% in GHGE and a median of 20–30%, when modelling shifts from typical Western diets to more environmentally sustainable dietary patterns.

The few reports on intervention studies suggest significant reductions in climate impact from a shift towards more plant-based protein sources (72, 76). More RCTs are needed on the implementation of sustainable plant-rich diets that can ensure adequate micronutrient intake.

It is noteworthy that also average current diets in the Nordic countries may be low in several nutrients. Recognising the unhealthy current diets together with population averages for specific nutrient falling below recommended reference values, and ensuring nutrient supply and health promotion have hitherto been the main focus of the FBDG. Also in the future, health evidence and nutritional adequacy in accordance with the revised dietary reference values of the NNR2023 at the population level should be the key constraints for developing national FBDG. The reduced content of animal products in total should include substantial reduction in red meat compared to current consumption level. Regarding fish intake, the focus should primarily be directed towards sustainably caught or sustainably farmed fish when recommending fish and seafood. At the same time, accompanying increase in plant-sourced foods should include a variety of vegetables and fruits, wholegrain products, pulses, nuts and seeds, and vegetable oils to ensure the intake of nutrients in a sustainable manner (53, 62). Studies, for example (40), suggest that women (relative to men) and vulnerable groups are more prone to experiencing significant changes in nutrient adequacy when it comes to dietary shifts. Ensuring nutritional adequacy when communicating sustainable FBDG involves not only focusing on reduction in meat, dairy and discretionary foods and beverages but also providing guidance in composing diets. Distinguishing vegetables and pulses in two food groups and specifying the quantity of pulses in a healthy diet may improve the overall communication.

It is well known that drastic reductions in all animal-sourced foods, such as a vegan diet or close to, may lead inadequate intake of critical nutrients such as vitamin B12, iodine, iron and vitamins D, and in some scenarios, vitamin A [e.g., (75)]. Dietary guidelines for these types of diets in all Nordic countries incorporate recommendations for dietary supplements and/or fortified foods and beverages. It is noteworthy that studies evaluating the nutritional and environmental effects of the current diet have mainly been conducted on adult diets. There is a lack of research data on vulnerable groups with increased demand for some nutrients. Such groups are children, pregnant and lactating women, adolescents and the elderly.

### Current FBDG and an approach for developing sustainable FBDG

The responsibility for delivering national sustainable FBDG to populations in the Nordic and Baltic countries rests with the national authorities. The Nordic countries have published FBDG for the general population as well as for specific population groups. Also, general guidelines regarding vegetarian diets are provided by the national authorities. All countries have used the NNR2012 as a basic reference and combined this with other country-specific reports. However, the degree to which environmental sustainability is included in current national FBDG differs between the Nordic countries, for example, the Norwegian FBDGs are solely health-based, and the Danish FBDGs have integrated sustainability in the quantitative guidelines. Whilst the overall qualitative guidelines are similar, only the Danish FBDG has a limit of total meat (of 350 g per week) and in addition separate quantitative guidelines on pulses and on nuts and seeds. Also, there are some differences between the quantitative guidelines for other food groups such as milk and dairy products, and total vegetables and fruits.

NNR2023 (12) offers comprehensive science-based advice on food consumption and dietary patterns, drawing from updated scientific health evidence, whilst considering the environmental impacts of various foods and diets, based on literature, that is, the present paper and other background papers (8–10). These together with the revised Nordic DRVs on nutrients should form the basis for national revisions of FBDG integrating environmental sustainability. Future development of FBDG may apply a combination of analyses with different approaches as presented in our paper and as defined by Perignon and Darmon (92). The framework of a five-step approach suggested by Wood et al. (62) is a recommended option in the national development process as it integrates the aspects of health, nutritional and environmental sustainability into intake values of food groups to provide quantitative guidance to the FBDG development. It offers a unique advantage: it outlines a diet that fulfils quantitative objectives for both health and environmental sustainability. This approach effectively integrates environmental considerations into sustainable FBDG development, employing an iterative modelling process that utilises the science evidence from NNR2023 and revised DRVs as targets. This not only offers guidance on what to eat but also quantifies the recommended amounts. Evaluation of the modelling results is crucial and should preferably include analyses of other dimensions of sustainability, for example, affordability and other socioeconomic impacts. The overall consequences of any proposed changes need to be carefully considered and possibly handled in other policy areas. Such an assessment must be done in a knowledge-based and transparent way.

### Strengths and limitations

The Nordic studies cover a broad spectrum of environmental sustainability. It is a strength that the studies cover the impact of total dietary patterns and the role of the food groups within the diets rather than comparison of impact of individual foods. However, more studies are warranted to better cover all Nordic countries and for the development of methods. In some of the reviewed studies, the nutritional adequacy of the current or proposed diet was not considered, and when included, the definition varied and often not consider the effects of bioavailability and other aspects impacting nutrient uptake and status. Moreover, health outcomes of food intake and varying proportions of energy-providing nutrients were not consistently considered.

Scenarios and modelling studies can illustrate consequences of changes in diets in the future. A limitation is that they rarely account for the impact of future technological advancements in food production and impact of large increases or decreases in production on the basic values of impact categories. The framework suggested by Wood et al. includes a way to take these issues into consideration (62).

Despite limitations in data, data for Nordic studies have expanded over the last decade (as indicated in Appendix B) and are sufficient to enable conclusions on required dietary changes to be drawn. However, environmental footprint data covering all aspects of environmental sustainability and the whole diet, for example, including both imported and local products, need continuous improvement to follow developments in the food system and to evaluate and maybe adjust to more specific dietary guidelines. In Nordic and Baltic countries, scientific uncertainties arise from limited data on environmental impacts (99) and dietary intakes (13). In addition, the published research focuses more on environmental aspects that are easily modelled (e.g., GHGE) and less on those that are not (e.g., eco-toxicity and impacts on biodiversity) (8). Although some studies show that the different impacts are related (97, 100), others find that this is not always the case (101, 102). Open access data, including metadata on biodiversity, carbon sequestration, climate impact and other environmental aspects of different stages of foodstuffs’ life cycles and different origin, as well as data on nutrient contents, would contribute to improve research that supports the national authorities’ guidance of the public. In addition, transparent information on the origin of the food and its components is needed since the environmental impact of foods is closely associated with the primary production, processing and logistics (101, 103). More research focusing on the Nordic and Baltic countries is required to better quantify the full environmental impact associated with higher proportions of plant-sourced foods and to evaluate potential trade-offs with other dietary shifts. Participatory and transdisciplinary work, where knowledge is co-created through collaboration between researchers and non-academic stakeholders, markets and government institutions, serves as a fruitful endeavour in research on complex sustainability transition challenges (104, 105).

Irrespective of which modelling method is chosen, valid data are important. The choice of health data and data on food consumption, nutrient content and environmental impact is crucial to provide valid results and correct interpretations (106). More advanced optimisation modelling, taking co-production and bioavailability of nutrients into account, has been developed, but continuous improvements in methods and updated data along with changes in production and energy supply are needed.

#### Implementation perspectives

Ways to facilitate the implementation of dietary changes warrant more attention. A recommendation for the optimal ratio of animal to plant sources in the diet alone will not guarantee a transition to healthy and sustainable diets, as the choice of individual foods within the food groups is significant. For example, citrus fruits, coffee, tea and nuts are all plant-sourced but had a different impact on water use, P application and extinction rate (31, 44, 45), and different types of fish and seafoods have different environmental impact (Appendix B), why products from sustainably managed stocks are recommended. Also, knowledge may be needed about how to include pulses, nuts and seeds, whole grain products and a suitable variation of vegetables in tasteful meals. Although plant-based alternatives to cow’s milk may have lower environmental impacts (107–110), they are not interchangeable regarding all nutrients (111) and bioactive components (112). With micronutrient fortification, such as calcium, vitamin D and vitamin B12 in fortified soy and oat beverages, they may have contents of these nutrients close to those of dairy products (113). Plant-based alternatives to meat may also contain both beneficial and adverse nutritional and health effects (114, 115). Careful analyses are required regarding the potential nutritional contribution of these meat or dairy product replacers to the total diet, as well as regarding the potential health effects. In addition, current scientific knowledge on the environmental impacts of plant-based animal-food alternatives is based on a limited number of LCAs that often rely on a combination of secondary data and collected data at production scale or from pilot-scale production facilities (107, 116). For reliable dietary scenarios, LCAs should preferably include information on the environmental impacts of food fortification, food additives, industrial ingredients and dietary supplement production.

Finally, the transition to sustainable diets must be made affordable and desirable for consumers at the national level. Since the transition is urgent, monitoring and evaluation should go hand in hand with public–private partnership initiatives, campaigns and development and piloting of case-studies to facilitate the transition at consumer level and to involve various food system actors. In Nordic countries, we have already successfully applied several effective steering methods and means of action, such as lunches free-of-charge to all children in Finland and Sweden (72, 117, 118); national guidelines for meals in early childhood education and care, schools, youth education institutions and worksites; and successful experience in public–private partnerships in Norway (such as the partnership for a healthier diet and the partnership for reducing food waste) and Denmark (e.g., in promoting whole grain in Denmark (119). Although interest amongst consumers and restaurants for information on sustainability already exists (120), and attitudes are set for the transition (21), clearer advice is necessary (33). The main challenges are to continuously produce sufficiently reliable background data to raise and keep awareness of consumers, the food industry, restaurant sectors and retailers and to make them capable to make informed choices for healthy and sustainable diets. Sustainability is a complex concept with several multilevel dimensions. Considering food production alongside consumption is necessary for a comprehensive assessment of environmental sustainability, as discussed in NNR2023 sustainability papers (8–10). These challenges are related to social sustainability, discussed in more detail in the NNR sustainability paper by Jackson and Holm (11).

## Conclusions

Climate change and biodiversity loss are threats to human well-being and planetary health, and there is a rapidly closing window of opportunity to secure a liveable and sustainable future for all (121). The Nordic studies conclude that animal-based foods, particularly meat and dairy products, are the largest contributors to dietary GHGE and land use in current diets. Modelling, optimisation and intervention studies confirm the potential of diet change to reduce not only negative environmental impacts of food consumption, in particular GHGE, but also cropland use, nitrogen application, phosphorus application, water use and biodiversity loss. There is a call for more comprehensive data, covering all aspects of environmental sustainability and the whole diet. Although scientific uncertainties exist, data are sufficiently strong to recommend a shift to a more plant-based diet. The shift from predominantly animal-based to predominantly plant-based diets needs to be larger than the previous (mainly health-based) FBDGs have recommended. Sustainable plant-based diets can be characterised as high in a variety of vegetables, fruit and berries, potatoes, cereal products as mainly whole grain products, vegetable oils, legumes (pulses), and nuts and seeds. They contain animal sources, such as fish from sustainably managed stocks, limited to moderate amounts of low-fat dairy and eggs and a limited amount of meat, with particular constraints on ruminant and processed meats. In addition, the content of discretionary food and drinks (e.g., sugar-sweetened beverages) should be limited. Exploring positive and negative implications of fortified foods and supplementation in contributing to nutrient intake, health and environmental sustainability may be needed. Dominantly or fully plant-based diets, such as vegan diets, require solutions besides specific dietary guidelines in terms of food fortification and/or dietary supplementation to ensure nutritional adequacy.

The goal at the national level might be to provide quantitative sustainable FBDG. Different types of studies or their combinations can be applied in a process of developing or adjusting sustainable FBDG at the national level. We recommend the five-step approach outlined in this paper, as it is a science-based, transparent and deliberated approach, involving experts on nutrition and health research and environmental and climate research as well as food systems stakeholders. Food and nutrition experts are needed both for conducting the modelling and for interpreting the results to ensure nutritional adequacy and positive health outcomes of dietary shifts.

## Appendix A

### Literature search and overview of the cited studies on environmental impact of dietary patterns in the Nordic countries

Web of Science searches for Nordic studies on environmental sustainability and whole diets were done in August 2022 with the following search strings: Diet sustain* ‘country’, for example, Diet sustain* Denmark. The search for Denmark was conducted on 19 August 2022 and resulted in 516 hits, 9 included measurements on both whole diet/food groups and any of the environmental impacts (GHGE, land use, water use, biogeochemical flows and biodiversity). The search for Finland was performed on 19 August 2022 and resulted in 290 hits, 10 included measurements on both whole diet/food groups and any of the environmental impacts. The search for Sweden was performed on 21 August 2022 and resulted in 526 hits, 12 included measurements on both whole diet/food groups and any of the environmental impacts. The search for Iceland was performed on 5 August 2022 and resulted in 34 hits, the search for Norway was performed on 5 August 2022 and resulted in 476 hits, the search for Estonia was performed on 5 August 2022 and resulted in 23 hits, the search for Latvia was performed on 5 August 2022 and resulted in 29 hits and the search for Lithuania was performed on 25 January 2023 and resulted in 42 hits. None of them covered estimations of the sustainability of the diets as a whole and any of the environmental impacts. Next, the core authors (ET, ME, HE, JM, HMM, ÞH and IÞ) reviewed country-specific results and complemented the search with national reports and supplementing studies. The studies on environmental impacts of dietary patterns, including the Nordic context and cited in the paper, are presented in Table A1 according to the research setting and methods used: i) observation studies, ii) modelling studies and iii) intervention studies. Other studies, including more recent studies, within the scope were referenced if they were relevant to the objectives of our paper. The manuscript was opened for public consultation, and new Nordic and Baltic studies were included if within the scope.

At the public hearing, we received comments from the following: Mikael Fogelholm (University of Helsinki), Martin Inderhaug (Animalia), Jenny Hagberg (Svensk Dagligvaruhandel), Chiara Lombardini (University of Helsinki), Satumaija Levón (Finnish Food and Drink Industries), S.Offe (ENSA – European Plant-based Food Association), Anna Maria Karlsen (NHO Mat og Drikke/FoodDrinkNorway), Anna-Lena Klapp (ProVeg International), Per Stålnacke (NIBIO), Cecilia Mille (Djurens Rätt), L.M. Granskog, Klas Ytterbrink Nordenskiöld et al. (Extinction Rebellion Sverige), Minna Kaljonen et al. (Finnish Environment Institute, University of Jyväskylä, National Institute of Health and Welfare), Johanna Kaipiainen and Charlotta Hyttinen (Finnish Vegan Association), Anna Richert (WWF Sweden), Ulrika Åkesson (Food Frame Sweden AB), Hildegunn Gjengedal (Norwegian Farmers’ Union), Puk Holm (Danish Agriculture and Food Council), Sara Sundquist (Swedish Food Federation), Sanna Kivimäki (Atria Oyj), Ellen Kathrine Ulleberg (Norwegian Dairy Council), Amanda Jakobsson (Svenskt Kött), Erica Lindberg (Svenska Fåravelsförbundet), Rune-Christoffer Dragsdahl (Vegetarian Society of Denmark), Kari Helen Bugge (TINE SA), Swedish Food Agency, Stella Höynälänmaa (Food Programme Director, WWF Finland), Ann-Kristin Sundin (LRF), Anna Jamieson (Sveriges Nötköttsproducenter SNP), Linda Cederlund [Kungl. Skogs- och Lantbruksakademien (KSLA)], Plant-food Sweden and Plantebranchen, Christiane Hoffmann (KLF), Hans Agné, and Merete Myrup Christensen (Danish Agriculture and Food Council –Dairy).

**Table A1:** Studies on environmental impacts of dietary patterns mainly conducted in the Nordic countries

Reference Country	Environmental data	Dietary data	Methods	Environmental variables	Dietary scenarios
Abadie et al. 2016 NO (27)	GHGE data from production, transport, preparation and waste	Norkost3	Average diet	GHGE	-
Bruno et al. 2019 DK (29)	LCA from-cradle-to-fork, average GHGE on international level	Food Balance Sheet by FAO	Pre-defined diet scenarios	GHGE	Standard Danish, carnivore, vegetarian and vegan
Colombo et al. 2019; 2020 Elinder et al. 2020 SWE (71, 72, 77)	LCA data on raw foods adjusted for waste (RISE)	Annual observed school food supply	Linear optimisation + school intervention	GHGE	- Lowest achievable GHGE - Minimised total relative deviation whilst imposing stepwise GHGE reductions, - Further constrained for deviations of pair-wise ratios of 15 food groups
Hallström et al. 2021 SWE (24)	Climate data based on LCA studies of foods representative for production of foods available on the Swedish market.	Dietary intake from two population-based cohorts, by FFQ	Analysis of dietary GHGE from different foods and by gender and age groups	GHGE	Current diet only
Hallström et al. 2022 SWE (31)	LCA: primary production, processing, packaging, international transportation and edible food loss and waste along the food chain (including consumer waste)	Swedish Mammography Cohort and Cohort of Swedish Men, FFQ	Observed diets	GHGE, land use, nitrogen and phosphorus application, consumptive water use, extinction rate, planetary boundaries	Current diet only
Hjort et al. 2020 SWE (39)	LCA from published literature, system boundaries: primary production up to and including the retail phase	Västerbotten Intervention Programme (VIP), FFQ	Observed diets	GHGE	Observed diet in 1996 and 2006
Jalava et al. 2014 FIN (70)	Water footprints of the crops: Mekonnen and Hoekstra 2011b, and animal products: Mekonnen and Hoekstra 2010.	FAO food balance spreadsheet (2013).	Modelling study	Water use	Diet scenarios: - Recommended diet (RD) – stepwise protein from animal product reduction diets: A50, A25, A12.5 and A0.
Kyttä et al. 2023 FIN (43)	Two LCA methods (Kuipers et al. 2021; Chaudhary and Brooks 2018)	FinDiet 2017 survey (Valsta 2018); DIPP cohort, 2008 (Kyttälä); Adolescents survey2008 (Hoppu); Health2000 survey (Montonen); Food Balance Sheet 2016 (LUKE); Fineli food composition database 2018.	Modelling study	Biodiversity impact Land use	Four scenarios: - Meat reduced to half - Meat reduced to one-third - Meat replaced with fish (including milk) - Vegan diet
Mazac et al. 2022 FIN (66)	Environmental impacts: AGRIBALYSE and World Food LCA Database.	European food consumption database (EFSA). 2013 compiled from 34 national food consumption surveys (n = 66,492 individuals) in 22 European Union countries.	Modelling study	GHGE, land use, water use	Modelled diets: - Omnivore (animal-based foods allowed in modelling) - Vegan - Novel and Future food (novel and future foods such as insects and ovalbumin allowed in the model) - All diets modelled in three models: minimised GHG, land use and water use.
Meinilä et al. 2022 FIN (35)	GHG data LUKE/Hartikainen et al. (unpublished)	Automatically accumulating food purchase data of loyalty card holders, *n* = 22,860. Nutrient content from Fineli.	Observed food purchase patterns by factor analysis, regression analysis to study the association between the patterns and GHG of the total purchases.	GHGE	Food purchase patterns based on 56 food groups - Traditional - High-energy - Plan-based - Animal-based - Ready-to-eat - Easy cooking
Martin and Brandão, 2017 SWE (54)	Databases Ecoinvent and AGRIBALYSE was complemented with LCA peer-reviewed studies, whilst data on import, export and waste came from FAO balance sheets.	FAO Food Balance Sheets, 2011	Modelling study	GHGE, acidification, eutrophication, land use, toxicity and biodiversity	Dietary scenarios: - Dietary guidelines - Reduced meat consumption - Vegetarian diet - Vegan diet − 200% increase in organic food consumption - 100% organically produced foods - 30% increase in foods produced in Sweden
Mertens et al. 2019 DK (data) (37)	LCA from SHARP-Indicators Database	DANSDA 2005–2008	Observed/current diet	GHGE Land use	Danish average diet
Mertens et al. 2021 DK (data) (67)	LCA from SHARP-Indicators Database	DANSDA 2005–2008	Modelling study: optimisation using Data Envelopment Analysis (DEA) to benchmark diets for improved adherence to FBDG	GHGE	Diet scenarios: observed, most preferred, healthiest (Nutrient Rich Diet Score) and most environmentally sustainable (GHGE)
Mertens et al. 2020 DK (data) (68)	LCA from SHARP-Indicators Database	DANSDA 2005–2008	Modelling study: optimisation using Data Envelopment Analysis (DEA) to benchmark diets for improved adherence to FBDG	GHGE	As above but all substituted by similar use meat replacers with and without fortification
Mittenzwei et al. 2020 NO (49)	LCA from different Norwegian sources	Grønlund, A. 2015. NIBIO Rapport 14 (2015). Norkost 3, Helsedirektoratet. 2015. Norkost 3. 18–70 år, 2011-11. Rapport IS-2000. Helsedirektoratet. Oslo.	Diet scenarios	CO_2_eq	Different scenarios based on different changes in meat contents and adherence to Norwegian FBDG
Moberg et al. 2020 SWE (30)	LCA data of impacts from cradle to retail in Sweden were summarised in a database (Moberg, 2019). Sources were, for example, World Food LCA database, Ecoinvent database, peer-reviewed LCA studies and LCA industry reports. Water use from WaterStat database.	Average direct consumption 2011–2015 (Swedish Board of Agriculture, 2019)	Calculated the impact of current data and benchmarked it against the planetary boundaries as they are presented in Eat –*Lancet.*	GHGE, land use N-application, P-application, Freshwater use Extinction rate	Impact of current diet only
Mogensen et al. 2020 DK (34)	For beef: LCA from cradle until exiting slaughterhouse and from slaughterhouse until ready-to-eat. For other foods: literature of LCA studies from cradle to ready-to-eat, including transport, packaging, storage, food waste and cooking.	DANSDA 2005–2008	Observed/current diet	GHGE Land use	Dietary patterns by principal component analysis: - Traditional - Fast food - Green - High beef
Natural Resources Institute 2021 ScenoProt study FIN (76) (See also Päivärinta et al., 2020 (73) Itkonen et al. 2021 (74) Pellinen et al. 2022 (75))	LCA (Hartikainen unpublished/Natural Resources Institute FIN)	3 × 4-day food records, Fineli food composition database	Intervention study	GHGE	Three diets with different ratios of animal to plant protein sources: 70:30, 50:50 and 30:70
Nordman et al. 2023 DK (69)	Data from Trolle et al. 2022	Dietary survey data, Danish adults	Quadratic modelling	GHGE	Optimised healthy and nutritionally adequate diet GHGE and departure from current diet
Risku-Norja et al. 2007 FIN (60)				GHGE land use, acid emissions, fuel and electricity consumption, the total material requirement of the diets	Four scenarios: - a nutritionally balanced diet, - a mixed diet based on dairy cattle with no pigs and poultry - a fish-vegetarian diet in which meat and eggs were substituted with vegetable products - as the mixed diet but based on 50% organic production
Röös et al. 2020 SWE (58)	LCA studies	Meat intake from Riksmaten 2010–11 (Swedish Food Agency)	Comparison of current consumption with an intake scenario	GHGE Land use	Dietary scenario: - Meat consumption reduced by 50% and replaced with legumes grown in Sweden
Saarinen et al. 2023 FIN (25)	See Appendix 1 in End report of the FoodMin project	FinDiet 2017 survey (Valsta 2018); DIPP cohort, 2008 (Kyttälä); Adolescents survey2008 (Hoppu); Health2000 survey (Montonen); Food Balance Sheet 2016 (LUKE); Fineli food composition database 2018.	Modelling study: FoodMin dietary model (LUKE) EnVit model (Seppälä ym. 2009, Nissinen ja Savolainen 2019).	GHGE Eutrophication Food waste	Four scenarios: - meat reduced to half - meat reduced to one-third - meat replaced with fish (including milk) - vegan diet
Sandström et al. 2017 FIN (45)	Land use estimated as country, crop and time specific yields from FAO 2016. Blue water use estimated from crop and country specific water use coefficients from Mekonnen and Hoekstra, 2010	FAOSTAT commodity balances (FAO, 2016), domestic and traded commodities (traded feeds included).	Study on the role of traded commodity supply (observed supply)	Land use (area), blue water use	Observed commodity supply with special interest in traded commodities
Saxe et al. 2013 DK (38)	LCA Food Database (2004) http://www.lcafood.dk and supplementary data from the literature		Modelling study; consequential Life Cycle Assessment.	GHGE	Average Danish Diet (ADD); diet based on the NNR; New Nordic Diet (NND)
Springman et al. 2018 GLOBAL (61)	Global country and crop-specific environmental footprint data (described in Harwatt et al. 2023)	- Country-specific food availability data (FAO) adjusted for food waste	Global modelling study	GHGE, cropland use, freshwater use and nitrogen and phosphorus application	- Reduction of animal-source foods following environmental objectives - Improving calorie intake and weight levels following food-security objectives - Using balanced diet patterns following public health objectives: flexitarian, pescatarian, vegetarian and vegan
Springmann et al. 2020 GLOBAL (33)	Global country and crop-specific environmental footprint data (described in Harwatt et al. 2023)	National FBDG (FAO), FAO’s food balance sheets adjusted for the amount of food wasted at the point of consumption.	Modelling study	GHGE, cropland use, freshwater use and nitrogen and phosphorus application	- National food-based dietary guidelines - EAT-Lancet guidelines - Pesco-vegetarian diet - Vegetarian diet - Vegan diet
Strid et al. 2019 SWE (42)	LCA from published literature, system boundaries: primary production up to and including the retail phase	Västerbotten Intervention Programme (VIP), FFQ	Sociodemographic differences in observed diet’s GHGE	GHGE	Current diet
Sundin et al. 2021 SWE (50)	LCA, system boundaries: from primary production up to industrial processing, excluding packaging, emissions from land-use change (RISE Food Climate Database, Florénetal 2017)	Overweight and obesity statistics of Sweden (SCB 2019), from which excess body fat and excess energy intake were estimated	Observed excess body fat and excess energy intake	GHGE	Current excess energy intake
Trolle et al. 2022 DK (28)	Data compiled by Aarhus University and DTU: Bottom-up approach: LCA from literature. And data from the Big Climate Database, CONCITO and 2.-0 LCA Consultants: Top-down hybrid approach.	DANSDA (2011–2013)	Pre-defined diet scenarios	GHGE	Danish Adapted Plant-Rich Diet, adherent to the EAT-Lancet reference diet
Valsta et al. 2022a FIN (In: Kaljonen et al. 2022) (40)	LCA (Seppälä et al. 2009)	FinDiet 2017 (Valsta 2018), Finnish nutritional recommendations (2014) Nutrient adequacy measured by a nutrient index with 10 components (Valsta et al. 2022, Appendix 2)	Pre-defined diet scenarios and Modelling study	GHGE Arable land Economic sustainability: earnings	- The current nutrition recommendations - 1/3 or 1/2 of meat and dairy substituted with legumes, nuts and seeds, fish and plant-based dairy alternatives, and fruit and vegetables until 500 g/day of total intake
Vanham et al. 2017 FIN (56)	Water footprints from the literature: Mekonnen and Hoekstra, 2011a, Mekonnen and Hoekstra, 2011b, and for fish Pahlow et al. (2015).	For countries: FAO Food Balance Sheets/For cities: FINDIET 2007	Modelling study	Water use	Dietary scenarios: - Average observed over 1996–2005 - Healthy, containing meat - Healthy pesco-vegetarian-Healthy vegetarian
Vieux et al. 2018 European FIN/SWE (data) (65)	LCA from Hartikainen and Pulkkinen (2016)	National surveys: FinDiet 2012 (FIN); Riksmaten 2010 (SWE); INRAN-SCAI-2005 (IT); NDNS 20018–12 (UK); INCA2 2006–7 (FR)	Modelling study (linear programming by applying stepwise GHGE reductions)	GHGE	- observed diet - nutritionally adequate diet - nutritionally adequate diet with minimised GHGE
Vieux et al. 2020 European FIN/SWE (data) (36)	LCA from Hartikainen and Pulkkinen (2016)	FinDiet 2012 Riksmaten 2010	Observed dietary clusters	GHGE	Six clusters from which one was identified as ‘more sustainable’
Wallén et al. 2004 SWE (57)	LCA studies on energy use and CO^2^ equivalents emissions from publications and data from food industry.	Current food consumption, per capita (Statistics Sweden, 2001) Intakes in Dahlin and Lindeskog’s definition of a sustainable diet (1999).	Comparing current diet with scenario of sustainable diet by food group	GHGE	The sustainable diet scenario included higher consumption of pulses, vegetables, cereals, fruits, eggs and fish, whilst it simultaneously had lower consumption of meat and meat products, sweets, sugary drinks, cream, cheese and rice than the current diet
Wood et al. 2023 SWE (62)	Mainly data from Moberg et al. 2020, and 2019	Current average Swedish diet as Moberg et al. 2020 (Average direct consumption 2011–2015 [Swedish Board of Agriculture, 2019])	Current diet – and iterative modelling	GHGE, nitrogen and phosphorus application, consumptive water use, biodiversity loss and cropland use	Iterative modelled more healthy and sustainable diet
Wright et al. 2023 NO (55)	Database at University of Oslo, based on LCA data	Dietary survey from 2019, based on a food frequency questionnaire	Comparing current diet with modelled diets adjusted to 5.3 MJ/day	Global warming potential, freshwater eutrophication, marine eutrophication, terrestrial acidification, water use and land use	Modelled diets according to Norwegian FBDG and Eat-Lancet reference diet, adjusted to 5.3 MJ/day
Zucchinelli et al. 2021 DK (44)	Water footprint for crop and livestock production in every import country of origin from Mekonnen and Hoekstra (2010, 2011).	Food Balance Sheet (FAO, 2018), scientific literature and public health recommendations	Comparison of current consumption pattern with pattern scenarios	Total green and blue water footprint and blue water-scarcity footprint	Current average Danish diet, and carnivore, vegetarian and vegan scenarios

## Appendix B

### Environmental impact data

This paper concentrates on Nordic dietary pattern studies. These studies use data from different sources, mostly national or Nordic data, but also international sourced data. As shown by Poore and Nemecek, a given food or food group can have widely different estimated environmental sustainability impacts, depending on how and where it has been produced (101). Analyses on a comprehensive global data set, with the system boundaries from farm inputs to include retailers level, on five environmental impact indicators (land use; freshwater withdrawals weighted by local water scarcity; GHGE; and acidifying and eutrophying emissions) showed the variation in impacts; for example, in GHGE (kg CO_2_eq/kg, IPCC 2007), medians (10 and 90 percentiles): bovine meat (beef herd) 51.7 (33.0, 180); bovine meat (dairy herd) 28.9 (15.8, 45.0); lamb and mutton 33.0 (21.3, 43.2); pig meat 9.8 (7.0, 21.4); poultry meat 7.8 (4.1, 19.9); milk 2.3 (1.5, 4.5); eggs 4.2 (2.9, 8.3). Ninetieth-percentile of GHGE and land use of herd beef were 12 and 50 times greater than 10th-percentiles of dairy beef impacts, respectively. Median GHGE values for plant foods were in general lower, for example, kg CO_2_eq/kg for potatoes 0.5; dry peas 0.8; tomatoes, onions and leek 0.7; root vegetables and brassicas 0.4; citrus fruit 0.3; apples 0.4; berries and grapes 1.4; and, finally, maize 1.2; oatmeal 2.6; rice 3.1. For wheat, maize and rice, 90th-percentile impacts were more than three times greater than 10th-percentile impacts on all five indicators (101). Poore and Nemecek also found high variation within and between protein-rich products in acidification, eutrophication and water use. However, for many products globally, correlations between indicators were low (0 to 30% in 26 of 32 impact–impact combinations assessed) (101). Clune et al. focused on global data on GHGE named global warming potential (GWP) (122). Data were based on many LCA studies (process based or economic input–output or hybrid LCA), and system boundaries were from farm to regional distribution centres. They also showed variation in values for the different food groups, where the lowest median GWP values were for field-grown vegetables and fruit, cereals (except rice) and pulses. Non-Ruminant meat, fish and cheese had medium GWP median values, whilst ruminant meat had the highest median GWP values (122).

The majority of studies estimating environmental impacts of food consumption used a bottom-up process-based LCA data (attributional LCA (a-LCA) (123), which takes into account, for a specific food, all processes within the defined system boundaries. Some databases are based on consequential LCA (c-LCA), which include consequences of changes in future consumption in the calculations and therefore will provide different impacts for some foods whilst other may be similar. Also, some databases use top-down approach and economic data for the different food sectors. Different approaches can provide differences in impact values for specific foods and influence conclusions based on calculations on the total diets (28, 124). As described in the EU PEF guideline (Product Environmental Footprint guideline) (125), many technical issues must be handled in the estimation of environmental and climate impact of food products. It is important to be aware of the system boundaries of the estimated values; often being from farm production to retailer level. Also, different allocation principles to allocate total impact to co-products from food production (such as beef meat and milk or wheat kernels and straw) may influence the specific impact values. Often economic allocation is used, but allocation may also be based on biological assessments (125). Another way of handling impacts of co-products is system expansion, where the impact is adjusted based on knowledge about other similar products that may substitute the co-product. Furthermore, the inclusion of the effect of land use changes (LUC) on the estimated GHGE may increase the impact values, and the total impact will depend on whether indirect or direct LUC (i-LUC or d-LUC) is used and even the method for calculation i-LUC (125).

The Nordic studies included in this paper use environmental impact values for individual foods from several LCA studies, for example, beef in Denmark by Mogensen et al. (126); pork in Denmark by Nguyen et al. (127) and Dorca-Preda et al. (128); fruits and vegetables in Denmark by Halberg et al. (129); vegetables in Sweden by Landquist et al. (130), milk in Sweden by Flysjö et al. (131), pork, poultry, eggs, milk and beef in Sweden by Cederberg et al. using a combined top-down and bottom-up LCA method (132); seafood in Norway by Ziegler et al. (133, 134) and meat analogues by Mejia et al. (135). Different data sets within the Nordic countries have been used, for example, the Swedish report by Fogelberg (4), the Swedish data sets from RISE (136) or Moberg et al. (30, 123) or Röös et al. (137), and the Danish data sets from Mogensen et al. (34) and the AU-DTU data (28), building on (34). In the cited Finnish studies, the environmental impacts of foods are mainly compiled by Natural Resources Institute Finland using their own databases and published literature. In Norway, a data set has been developed and used by Wright et al. (55), and a former data set was introduced by Abadia et al. (27). In Denmark, also the CONCITO database (The big climate database), based on a hybrid top-down, c-LCA method, has been available since 2021 and recently updated (138). An analysis by Mogensen et al. (139) compared the carbon footprint values (kg CO_2_-eq per kg food (edible weight)) of selected food items from different databases and showed, for example, differences in CF of beef (from 13.9 [Mogensen] to 24.9 (Moberg), 27 (RISE) and 50.4 (CONCITO [incl. i-LUC]); milk from 0.57 (CONCITO), 0.9 (RISE) to 1.16 (Mogensen) and 0.8–2.5 (Röös); pork from 4.1 (Moberg) and 4.3 (CONCITO) to 4.9 (DK, AU) and 6 (RISE); chicken from 2.4 (RISE), 2.57 (Moberg), 2.75 (CONCITO) to 4.9 (AU-DTU); and egg from 0.7 (CONCITO), 1.4 (RISE), 2.0–2.2 (Moberg) to 2.3 (Mogensen 2020) and 1.4–4.6 (Röös)). A report on foods on the Norwegian market (140) showed GHGE of meat from dairy cows being approximately 19.5 kg CO_2_-eq per kg product (range: 11–37.5), however estimated per kg carcass weight (incl. meat and bones). The report concluded that it is higher than similar meat from other European countries, for example, Denmark. Furthermore, it is concluded about foods on the Norwegian market that lamb and sheep meat emits 0.7 times more than beef; beef emits 4.5 times more than pork (range 3.5–8.5); and beef emits 8.5 times more than chicken (range 4.5–16) (140). These comparisons also indicate that GHGE of pork and chicken may be at the same level. A paper by international authors, including from Sweden (141), showed that GHGE per kg edible fish and seafood varied depending on the methods used for capture and farmed fish and seafood, GHGE of seafood being highest for shrimp, flounder and lobster and four-fold as high as from wild cod and haddock, but farmed bivalves and small pelagic fish having the lowest impact. GHGE by farmed salmon varied from being as low as cod to around three-fold as high, whilst wild salmon generally had impact similar to the lower end of farmed salmon (141). The highest nutrient benefit at the lowest emissions was found for wild-caught small pelagic and salmonid species and farmed bivalves like mussels and oysters (142). Other environmental impacts are included in Nordic data sets such as Potter et al. (land use, biodiversity impact from land use, total water use and regional impact of blue water use) (107), Moberg et al. (GHGE, land use N-application, P-application, Freshwater use and Extinction rate) (30, 123) and Wright et al. (global warming potential, freshwater eutrophication, marine eutrophication, terrestrial acidification, water use and land use) (55). Few studies compare impact of conventional and organic produced foods; however, GHGE per kg seems to be similar of most types of foods (whilst other impacts show different results (139, 143)). More studies that include several impact categories are warranted (144).

## Appendix C

### Denmark

- Eat plant-rich, varied and not too much
- Eat more vegetables and fruit
- Eat less meat – choose legumes and fish
- Eat whole-grain foods
- Choose vegetable oils and low-fat dairy products
- Eat less sweet, salty and fatty foods
- Thirsty? Drink water

In addition to a focus on healthy and climate-friendly food, the Danish Dietary Guidelines also encourage Danes to choose vegetables and fruit in season, to go for the environmentally friendly choice of fish and to reduce food waste (86).

### Finland

- Eat vegetables, fruits and berries frequently (a minimum of 500 g/day, excluding potatoes).
- Eat whole-grain cereals (bread, porridge, pasta, etc.) several times a day. Prefer fibre-rich and low-salt products. Avoid products made of refined flour with an abundance of hard fat and sugar.
- Use soft vegetable oil-based spreads on bread and vegetable oils in cooking and salads.
- Eat fish (of different kinds) two to three times a week.
- When eating meat, choose low-fat, low-salt products and limit the amount of red meat and meat products to <500 g a week.
- Consume fat-free/low-fat milk products daily (5–6 d /day) and two or three slices of low-fat cheese.
- Drink water for thirst. Decrease consumption of soft drinks and sweet juices.
- Use low-salt products (salt intake should be <5 g/day).
- Undertake moderate physical activity (brisk walking) for at least 150 min a week or hard physical activity (running) for 75 min a week.
- In addition, eat regularly. Read and learn to understand product labels.

The food-based guidelines provide examples of preferable sustainable food choices that are based on the sustainability section in NNR2012. They emphasise the consumption of vegetables, roots, potatoes, berries and fruits, domestic legumes and cereal products, and wild water fish and suggest favouring of rapeseed oil and margarine over other fats and oils and tap water over bottled water. It is also mentioned that beef production contributes the most to climate change and eutrophication; however, noting that adequate production of cattle is essential for milk production and its downstream products (83).

### Iceland

1. Focus on the diet as a whole
2. The importance of a varied diet.
3. Fruits and lots of vegetables (500 g a day, at least half of it should be vegetables, juice not included).
4. Whole-grain foods at least twice a day.
5. Fish 2–3 times a week, of which fatty fish once a week.
6. Eat meat in moderation, max 500 g of red meat/week.
7. Low-fat milk without added sugar, two portions a day.
8. Healthier fat (use oil instead of butter or margarine in cooking).
9. Reduce salt intake (max 6 g/day)
10. Reduce added sugar
11. Vitamin D – daily

The Keyhole – healthy choices made easy (84).

### Norway

1. Enjoy a varied diet with lots of vegetables, fruit, berries, whole-grain foods and fish, and limited amounts of processed meat, red meat, salt and sugar.
2. Maintain a good balance between the amount of energy you obtain through food and drink and the amount of energy you expend through physical activity.
3. Eat at least five portions of vegetables, fruit and berries every day.
4. Eat whole-grain foods every day.
5. Eat fish two to three times a week. You can also use fish as a spread on bread.
6. Choose lean meat and lean meat products. Limit the amount of processed meat and red meat.
7. Include low-fat dairy foods in your daily diet.
8. Choose edible oils, liquid margarine and soft margarine spreads instead of hard margarines and butter.
9. Choose foods that are low in salt and limit the use of salt when preparing food and at the table.
10. Avoid foods and drinks that are high in sugar.
11. Choose water as a thirst-quencher.
12. Be physically active for at least 30 min each day.

Look for the Keyhole when shopping for food (80).

### Sweden

- More vegetables and fruit – Eat lots of fruit, vegetables and berries! Ideally, choose high-fibre vegetables such as root vegetables, cabbage, cauliflower, broccoli, beans and onions.
- More seafood – Eat fish and shellfish two to three times a week. Vary your intake of fatty and low-fat varieties and choose eco-labelled seafood.
- More exercise – Exercise for at least 30 min every day! Take brisk walks, for example, and reduce the amount of time you sit still by taking brief, active breaks.
- Switch to whole meal – Choose whole-grain varieties when you eat pasta, bread, grain and rice.
- Switch to healthier fat – Choose healthy oils when cooking such as rapeseed oil or liquid fats made from rapeseed oil, and healthy sandwich spreads. Look for the Keyhole symbol.
- Switch to low-fat dairy products – Choose low-fat, unsweetened products enriched with vitamin D.
- Less red and processed meat – Eat less red and processed meat, no more than 500 g a week. Only a small amount of this should be processed meat.
- Less salt – Choose food with less salt. Use less salt when you cook but choose salt with iodine when you do use it.
- Less sugar – Hold back on the sweets, pastries, ice creams and other products containing lots of sugar. Cut back on sweet drinks, in particular.
- Maintain a balance – Try to maintain energy balance by eating just the right amount.
- The Keyhole – healthy choices made easy – Check for the Keyhole symbol. This is a National Food Agency symbol, which can help you to find food containing less sugar and salt, more whole grains and fibre, and healthier fat or less fat.

Food waste is only briefly mentioned in the FBDG, as there is ongoing parallel work with a national action plan to reduce food waste (85).
